# Perception and Use of Primary Healthcare Services Among People With Cardiometabolic Diseases in Two Resource-Limited Areas in Nepal: A Mixed Methods Study

**DOI:** 10.3389/fpubh.2021.698030

**Published:** 2021-09-23

**Authors:** Nicholas Peoples, Enying Gong, Kamal Gautam, Shree N. Khanal, Brandon A. Kohrt, Suraj Koirala, Archana Amatya, Shangzhi Xiong, Truls Østbye, Jeffrey Moe, Qian Long, Lijing L. Yan

**Affiliations:** ^1^Baylor College of Medicine, Houston, TX, United States; ^2^Global Health Research Center, Duke Kunshan University, Kunshan, China; ^3^Melbourne School of Population and Global Health, University of Melbourne, Melbourne, VIC, Australia; ^4^Transcultural Psychosocial Organization Nepal, Kathmandu, Nepal; ^5^George Washington University, Department of Psychiatry and Behavioral Science, Washington, DC, United States; ^6^Institute of Medicine, Department of Community Medicine and Public Health, Tribhuvan University, Kathmandu, Nepal; ^7^Duke Global Health Institute, Duke University, Durham, NC, United States; ^8^School of Health Sciences, Wuhan University, Wuhan, China

**Keywords:** primary healthcare (PHC), cardiovascular disease, hypertension, stroke, diabetes, Nepal

## Abstract

Nepal is a country in south Asia with a high burden of cardiometabolic diseases (CMDs). Strengthening primary healthcare (PHC) is a key strategy to mitigate this increasing burden and achieve universal health coverage. While previous studies in Nepal have assessed PHC use among the elderly, none have specifically explored PHC use among people with CMDs. Therefore, this mixed-methods study aimed to assess the use and perception of PHC services in Nepal among people living with CMDs for primary and secondary prevention of cardiovascular disease. We used a quantitative survey followed-up by semi-structured qualitative interviews. The sampling frame comprised five PHC facilities in Sindhuli district (rural; eastern Nepal) and five in Kailali district (urban; western Nepal), with participants selected from each facility via convenience sampling. 114 people (mean age: 54.5 ± 14.7, sex ratio 1.04) with CMDs participated in the survey. Survey data showed general dissatisfaction with PHC services. Medicine cost was rated “too expensive” by 52 and 63% of rural and urban participants, respectively. Interview data showed that perceived poor bedside manner was tied to negative perceptions of PHC quality, and vice versa. Lack of resources and excessive barriers to care was mentioned by every interviewee. In conclusion, PHC use was high but overall satisfaction relatively low. Our results suggest that bedside manner is a practical target for future research. Additionally, we identified several barriers to care, and, based on existing literature, we suggest electronic-health interventions may have potential to mitigate these challenges.

## Introduction

Control of cardiometabolic diseases (CMDs), which encompasses cardiovascular disease (CVD, primarily heart disease and stroke) and related conditions such as hypertension and diabetes, is an urgent global health priority (1–5). Primary healthcare (PHC) is on the frontline of CMD control because it targets modifiable risk factors, promotes healthy lifestyle habits, and provides continuity of care across the lifespan (6–10). PHC utilization is a key indicator of PHC accessibility, affordability, and perceived effectiveness (11–14). Since prevention and control of CMDs depends heavily on mobilizing health systems to serve large populations, PHC utilization can also be viewed as one indicator for the potential of PHC to combat CMDs (15, 16). Likewise, understanding patient perception of PHC use elucidates factors which may influence overall utilization, thereby providing meaningful targets for future research and intervention (13, 17–19). Despite its strategic position to combat CMDs, however, primary healthcare is often the weakest link in already underdeveloped health systems in many low- and middle-income countries (LMICs) (20–26).

This study examines the use and perception of PHC services for the prevention and management of CMDs in Nepal, a small, landlocked country in south Asia. Nepal is a low-income country with about 80% of the population living in rural areas. It is considered to be one of the least developed countries globally, with ~15% of the population below the international income poverty line (27). Non-communicable diseases (NCDs) account for more than 44 % of deaths and 80 % of outpatient contacts in Nepal (28). Tertiary care is limited, expensive, and disproportionately concentrated in urban areas (29). In contrast, by law, basic health services must be provided free of charge within PHCs (30). Though Nepal is well-positioned to benefit from strong PHC services for CMD control, literature on the perception and use of these services in Nepal is sparse. Studies have examined PHC utilization among aging Nepalese populations (29, 31, 32), but not specifically people with CMDs. Therefore, a better understanding of these factors in Nepal may illuminate the potential of PHC in both prevention and management of CMDs in a challenging setting.

Our primary study objectives are to assess the use and perception of PHC services for prevention and management of CMDs among people living with CMDs in Nepal. We explore PHC facilities in both an urban and a rural setting and capture data on patient utilization of PHC services for both primary and secondary prevention of cardiovascular disease.

## Methods

### Overview and Study Setting

This study was conducted as part of the four-country “FAITH” (feasibility assessment of invigorating grassroots primary healthcare) study, whose protocol is published elsewhere (15). We used a mixed-method study design, conducting structured quantitative surveys followed up by a smaller number of qualitative in-depth interviews with select participants. Our sampling frame was ten PHC facilities across two regions of Nepal: five in Kailali district and five in Sindhuli district. Sindhuli is a rural, mountainous area in eastern Nepal which was severely affected by the 2015 earthquake. Kailali, in contrast, is a comparatively more urbanized district in the far Western development region. Topographically it is a flat plain with some hilly areas, and it shares a border with India. Facilities were selected by cluster convenience sampling.

### Participants

For the quantitative survey, we used convenience cluster sampling to recruit at least ten participants with CMD from each PHC facility according to the following inclusion criteria: (1) over 18 years of age; (2) have ever been diagnosed with at least one of the following conditions: heart disease, stroke, hypertension, and/or diabetes; (3) willing to participate and able to reach the sampled PHC facility to be interviewed. Exclusion criteria included: (1) people who were seriously ill, and (2) people with communication difficulties who are unable to answer the questionnaires or participate in the interviews. To compensate for the inherent weaknesses in cluster convenience sampling, we applied additional selection strategies to each facility to achieve the most balanced and representative sample possible: (1) an equal ratio of male and female participants; (2) a balance of patients across age groups, including at least two participants younger than 45 at each facility; (3) when possible, two participants who had not obtained care from this facility in the previous 12 months; and (4) those who had been diagnosed by a healthcare professional as having heart disease and/or having experienced a stroke were prioritized over those with other conditions. Strategy 3 compensates for using the PHC facility as our sampling frame, where we are more likely to enroll active PHC patients. Strategy 4 corrects for the higher proportion of hypertension/diabetes cases which would otherwise comprise most of the sample.

For the qualitative component, we followed up a smaller number of participants from the quantitative survey for inclusion in the in-depth semi-structured interview. Data collectors selected two participants at each facility to be interviewed, based on willingness to participate and ability to communicate. Our target was 10 interviews from each study district and 20 overall, as this number is usually enough to reach information saturation.

### Data Collection

Our quantitative questionnaire was modeled after the WHO STEPS survey (33) and included four sections: demographics, cardiovascular disease history, access to PHC services, and medication history. We measured “PHC use” as a function of visits within the past 12 months and “time needed to see a provider” as the combination of travel time and wait time. We measured “perception” by using Likert-scale ratings of travel time, wait time, medicine cost, and overall treatment cost (the full sum paid by the patient to the PHC for their visit). For the qualitative component, we developed a standardized interview guide of five questions for the semi-structured in-depth interviews. Questions covered disease management, satisfaction with PHC services and treatments, and emergency preparedness. Interviews were conducted in Nepali language, audio-recorded, and translated to English.

### Data Analysis

Quantitative data analysis was completed via R Studio (34). Descriptive analysis of sociodemographic factors included age, gender, education status, health status, occupation, and marital status. For subgroup analysis, we dichotomized participants by location (rural/urban) and disease status (hypertension/diabetes vs. CVD), using Kruskal-Wallis tests for continuous variables (number of PHC visits, travel time, and wait time) and Pearson's Chi-Squared for categorical variables (Likert-scale ratings of travel time, wait time, medicine cost, and treatment cost).

Qualitative data analysis was completed using NVIVO 11 (QSR International). A team of three trained coders, using a thematic analysis approach, created the codebook and systematically coded all 20 in-depth interviews to identify relevant themes and sub-themes. The codebook was drafted and finalized based on discussion and mutual agreement of all three coders, and an inter-rater reliability of >80% was achieved prior to the start of coding. Theme frequencies and key themes mentioned by interviewees from each district were tabulated (**Table 3**). Finally, representative quotes from the major thematic areas were selected to illustrate participants' attitudes and perceptions of receiving care at their local PHC facility.

### Ethical Approval

We received ethical approval from the Duke University Institutional Review Board (IRB) (Protocol Number: Pro00082962) and the Nepal Health Research Council (NHRC) (Reg. No. 158/2017). We obtained written informed consent from all participants prior to starting data collection.

## Results

### Quantitative Data

Table 1 shows the sociodemographic profile of our participants. We completed 114 valid questionnaires and 20 in-depth semi-structured interviews in August 2017. Of those, 63 surveys and 10 interviews came from urban areas (Kailali district) and 51 surveys and 10 interviews came from rural areas (Sindhuli district). The mean age (±standard deviation) was 54.5 ± 14.7 years and the sex ratio was 1.04. In both study locations, sociodemographic variables indicated low socioeconomic status among participants. In Sindhuli, 70% of participants were illiterate or educated below a primary school level; the most common form of employment was agriculture (45%); unemployment was 6%; and the most common self-reported health status was “bad/very bad” (45%). In Kailali, 56% of participants were illiterate or educated below a primary school level; the most common form of employment was agriculture (37%); unemployment was 16%; and the most common self-reported health status was “average” (37%).

**Table 1:** Participant sociodemographic profile.

	**Urban** ***(n*** **= 63)**			**Rural (*****n*** **= 51)**	
	***n***	**%**		***n***	**%**
**Age (years)**					
<45	20	32		13	26
45-65	24	38		23	45
>65	19	30		15	29
**Sex**					
Female	31	49		25	49
Male	32	51		26	51
**Education Status**					
Illiterate	15	24		20	39
Literate	20	32		16	31
Primary Level	8	13		3	6
Secondary Level	10	16		9	18
Higher Secondary	9	14		1	2
Undergraduate	1	2		2	4
Post-Graduate	0	0		0	0
**Marital Status**					
Currently married	58	92		45	88
Never married	1	2		1	2
Widowed	4	6		4	7
Divorced/separated	0	0		1	2
**Self-Reported Health**					
Very good/good	19	31		13	25
Average	23	37		25	29
Very bad/bad	20	32		23	45
**Occupation**					
Government	5	8		2	4
Agriculture	23	37		23	45
Business	10	16		7	14
Student	0	0		1	2
Housewife	13	21		4	8
Retired	2	3		3	6
Unemployed	10	16		3	6
Other	0	0		8	16

Table 2 describes annual PHC use, travel time, and wait time, and compares differences between two subgroups: location and disease type. PHC use was frequent to semi-frequent, with the mean number of PHC visits in the past year being 7.5 ± 6.6 in urban areas and 8.7 ± 9.5 in rural areas. This is an average of one PHC visit per every 1.4 to 1.6 months. There were no significant differences between subgroups except for travel time by disease status, with the CVD group reporting longer travel times to reach their local PHC facility.

**Table 2:** PHC Utilization and Time Needed to See a Provider.

	**Location**			**Disease Type**		
	**Urban**	**Rural**	***p***	**CVD**	**Hypertension/Diabetes**	***p***
	*n* = 63	*n* = 51		*n* = 50	*n* = 62	
**PHC Visits Past Year**						
Mean	7.5	8.7	0.500	8.1	8.2	0.468
SD	6.6	9.5		8.9	7.4	
**Travel Time (min)**						
Mean	14.5	21.8	0.106	22.1	13.1	0.002
SD	14.7	24.6		22.0	13.8	
**Wait Time (min)**						
Mean	11.3	15.4	0.180	13.1	12.7	0.383
SD	9.2	24.5		9.5	12.4	

**Figure 1:**
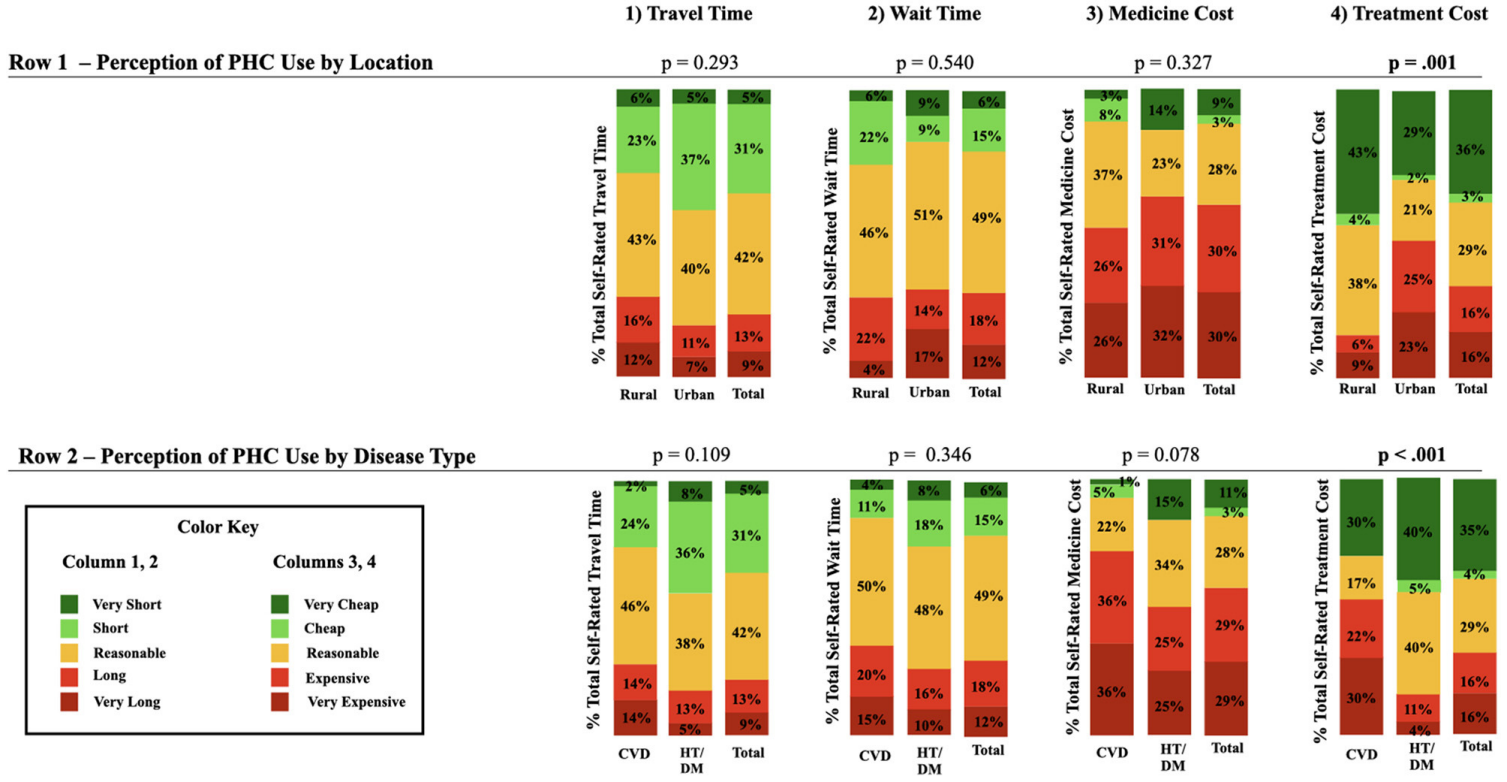
Perception of PHC use.

Figure 1 describes perception of travel time, wait time, medicine cost, and treatment cost, with subgroup analysis by location (urban vs. rural) and disease type (CVD vs. hypertension/diabetes). Treatment cost was found to be significant (*p* ≤.001) in both subgroups, with a higher proportion of both urban participants and participants with CVD reporting treatment cost as “too expensive” compared to rural and hypertension/diabetes participants. The difference in treatment cost can be visualized in column 4. Additionally, column 3 shows that the highest amount of dissatisfaction was with respect to the cost of medicine. Medicine cost was rated “too expensive” by 72% of participants with CVD, 50% of those with hypertension/diabetes, 52% of rural residents, and 63% of urban residents.

### Qualitative Data

Table 3 shows the results of the thematic analysis for the 20 in-depth interviews. We identified three major themes: barriers to PHC utilization, perception of PHC services, and participant recommendations. We further identified five to eight sub-themes within each major theme. Within the following text, numbers within brackets signify the proportion of respondents who mentioned the respective topic (e.g., 100% = all participants mentioned / said this). A sociodemographic profile of interview participants is included in Appendix.

**Table 3:** Summary of Thematic Analysis.

**Themes and Subthemes**	**Times Mentioned**		**Participants Discussing** **Theme**		**Key Points**
	**Urban**	**Rural**	***n*** **= 20**	**%**	
**A) Barriers to PHC Utilization**					
Financial	18	34	14	70	Patients use PHC centers because they are the closest point of contact with the health system, and hospitals / tertiary care is too far away. However, despite visiting a PHC center, patients cited lack of machines, doctors, medicine, and cost of treatment as barriers to receiving proper care or returning to the PHC center.
Lack of Resources	44	54	20	100	
Geographic / Travel-related	12	11	14	70	
Education	1	2	3	15	
Medicine Not Effective	2	4	5	25	
Distrust Health Workers	0	3	2	10	
Poor Administration / Leadership	0	3	3	15	
Insufficient Open Hours	2	0	1	5	
**B) Perception of PHC services**					
Wait Time	2	1	3	15	High emphasis placed on physician behavior/competency over other areas. Perceived quality of medical care was strongly tied to perceptions of staff behavior / bedside manner as gentle and caring or not.
Cleanliness	0	1	1	5	
Staff Behavior	10	9	9	45	
Health Worker Competency	8	7	15	75	
Treatment Satisfaction	2	6	8	40	
**C) Patient Recommendations**					
Medicine / Equipment Availability	25	27	20	100	Participant recommendations were consistent: availability of essential resources and financial assistance / reduced costs were the major suggestions to improve PHC quality.
Lower Physician Turnover /Absence	1	4	5	25	
Access to Specialist Care	18	9	10	50	
Free Medicine / Financial Assistance	12	35	17	85	
Health Education / Awareness	0	5	4	20	

When asked about barriers to PHC utilization, all participants expressed frustration with the lack of resources at their local PHC (100%), but most also noted that traveling elsewhere for healthcare was not feasible due to distance, cost of travel, and difficulties associated with travel such as heavy seasonal rain and landslides (70%).

*“For a heart patient like me, if there were services like ECHO, ECG, medicine, and a doctor available here at the PHC itself, we needn't go elsewhere…It would be easier if all things were available here itself.”* (Participant SS, Sindhuli District)

Despite a national policy that all PHC centers should have a doctor, many participants reported this was not the case. (When a physician is not present, health assistants and auxillary healthcare workers generally provide care).

*“I hope doctors will be available soon. The worst thing is, we don't have MBBS doctor here. He comes once in a while, stays for 1–2 days and leaves. This the main problem: patients have to bear it. Doctors don't stay here.”* (Participant HS, Kailali District)

When asked about perception of the PHC center, participants emphasized staff behavior (45%) and their competency (75%). While assessments of provider competency were mixed, these appraisals (either positive or negative) were strongly tied to perceptions of the physician's attitude and bedside manner.

*“The most recent place I went for check-up, I really liked the service and attitudes of the (female staff) there. And the doctor. his way of talking and checking the patients was really very good. The place I went before there was horrible. The doctor was rude and I felt that they would certainly kill most patients just by talking to them”* (Participant DN, Kailali District).

One common point of dissatisfaction, mentioned by both urban and rural residents, was young doctors (either recently graduated or still in training) staffing PHC facilities. They were perceived as being both less competent and less likely to stay and practice at the PHC facility than other physicians.

*“We don't have good doctors here. Only the learners come here. They come here for internship and for practical. Good doctors don't come here and I don't like it”* (Participant MS, Kailali district).

Conversely, having staff from the community was perceived more favorably.

Finally, recommendations for improved PHC services were consistent. Every interviewee suggested improving the availability of equipment and essential medicines. One participant concisely summarized the dilemma between close but low-quality PHC care and distant, higher quality tertiary care:

*“As per my knowledge, it will be better if we take the patient to the Belghari PHC first as this PHC is very close and in our community. But for us to be able to choose the Belghari PHC first, we need equipment and facilities in here”* (Participant JR, Sindhuli District).

Health education and counseling, an area where PHC is uniquely positioned to excel, was also noted as being substandard:

*“Most of the people are unaware and don't have any idea how to manage these kind of disease. As the awareness about the proper care of the heart is absent in our community, priority should be given to these things…There was one member of our community who died when he took two pills at once that was meant for 2 days as he had forgot to take the pills the day before. He was immediately taken to Kathmandu but he died anyway.”* (Participant JR, Sindhuli District).

## Discussion

### Interpretation of Data

This study examined PHC use and perceptions among people with cardiometabolic disease in Nepal. Global literature has described PHC use and patient satisfaction in many contexts, and previous studies in Nepal have focused on PHC use among aging populations (29, 31, 32). As the first study (to the best of our knowledge) to specifically describe use and perception of PHC services for CMDs in Nepal, this manuscript serves as a call to action for contributing new knowledge to this important space in the literature.

In our study, though PHC use was high, satisfaction was relatively low. Moreover, our result for mean PHC use may be an underestimate among PHC users given that our sample comprises both current PHC users (*n* = 101, 91.4%) and participants who had not sought care a their PHC in the past 12 months (*n* = 11, 9.6%). At face value, this may seem paradoxical since other studies have found that perception of low quality and unavailability of needed services is associated with lower PHC use (13–15). The difference in our study may be explained in the following way: we specifically studied PHC use among people with CMDs, a group that requires regular checkups and longitudinal management of their health condition(s), while other studies examined PHC use among whole communities for a broad variety of conditions. Due to the chronic nature of their illness, people with CMD may be more likely to consistently use local PHC facilities. This is in contrast to those with acute problems or other medical issues where a once-off visit to a more distant tertiary care facility may be judged the better option. This indicates that even though PHC facilities are not functioning at a level to meet patient expectations, they are still the first choice treatment center and on the frontline of CMD management for this group. Strengthening CMD prevention and management in local PHC centers therefore still offers strong potential to mitigate the burden of CMDs within their respective communities.

Medicine cost received the highest dissatisfaction ratings from both rural and urban residents. This represents a serious challenge, as many drugs for the prevention and management of CMDs are classified as “essential medicines” which the WHO recommends should be available “at a price the individual and community can afford” (35). In Nepal, the essential medicines policy goes even further: the essential drugs list for PHC contains 60 medications which are required to be available *free of charge* at governmental health facilities (36). Despite this, medication costs are the highest household health expense and a major driver of impoverishment in most low-income countries like Nepal (35). As 60% of participants rated medication cost “too expensive” and 85% recommended decreasing medication cost, disparity between policy and practice likely persists. Moreover, despite that WHO PEN (package of essential NCD interventions) recommends that essential medicines should be available at PHC facilities (37), 100% of our interview participants recommended increasing medicine availability, implying at least some needed medicines were not available. While resource constraints are an expected issue, it is also estimated <10% of PHC facilities in Nepal are in compliance with required drug storage practices for the essential medicines they did receive (36). This contributes to drug degradation and further perpetuates the scarcity of essential medicines. Therefore, our results suggest more attention should be given to closing the gap between policy and practice, ensuring that qualifying essential medicines are both available and free of charge at PHCs in Nepal and confirming the essential drugs list adequately covers CMDs.

The interview data adds nuance to the discrepancy between high PHC use and lower satisfaction ratings. In large part, participants attributed the higher use of local PHC services to financial, logistic, and geographic barriers to travel elsewhere for care. This helps explain our survey finding of high PHC use among our sample of people with CMDs in tandem with low satisfaction ratings. Negative perception of PHC services was most strongly driven by perceived lack of needed medicines, equipment, and health personnel. These negative perceptions were mitigated, in part, when participants believed their healthcare providers had good bedside manner. This mediating effect of provider behavior has been documented elsewhere, for example in a similar study on patient perception of PHC in India (19). That study observed that patients placed high emphasis on physician behavior and the patient-physician relationship as strong indicators of satisfaction. Our study results are congruent with these findings, and indicate that bedside manner and the provider-patient relationship are key factors for PHC use among patients with CMDs. A tool has been developed in Nepal for observed rating of communication skills and therapeutic relationships between patient and provider (38, 39), as well as patient-rated version of interactions with providers, which is correlated with mental health outcomes in PHCs (40). In addition, the WHO Foundational Helping Skills training, which focuses on provider communication skills, is currently being piloted in Nepal (41). The findings here support the need for structured evaluation and training in provider communication skills, such as combining WHO Foundational Helping Skills training with WHO PEN training.

### Strengths and Limitations

This study is the first to examine perception and use of PHC services for CMDs in Nepal. The use of mixed-methods bolstered the overall quality and breadth of data, and we explored utilization of PHC services for both primary and secondary prevention of cardiovascular diseases. Additionally, sampling facilities from urban and rural areas adds to the strength of our study.

The main limitations are the use of only two districts of Nepal as study sites, the use of cluster convenience sampling, and the limited sample size. With respect to study sites, Nepal is an extremely diverse country, both ethnically and geographically. Within the limitations of being only two study sites, Sindhuli and Kailali do portray characteristic regions of the country and serve for our purposes as an exploratory study. Second, cluster convenience sampling was used out of necessity given budget and timing restraints. This method can potentially bias results via sampling bias. To mitigate this, we introduced additional inclusion/exclusion criteria such as gender parity, age parity, parity between CMD type, and people with CMDs who had not used the PHC facility within the past year, to reduce the inherent limitation of cluster convenience sampling. Finally, while our sample size of 114 participants is too small to act as a regionally or nationally generalizable sample, the number was large enough to permit statistical subgroup comparisons. In the qualitative component, our interviewees reached information saturation before completion of the 20th interview, evidencing sufficiency of the sample size for our purposes. Taken together, these limitations do not invalidate our data. We are therefore able to draw conclusions appropriate to the level of an exploratory study, using our results to highlight evidence gaps and important areas of investigation.

### Recommendations and Future Research

Given the exploratory nature of this study, our recommendations focus on identifying the highest priority problems. From our mixed-methods approach, we found that PHC use was relatively high, while overall satisfaction relatively low. What can be done to improve satisfaction? Increase in the availability of essential medicines, diagnostic and therapeutic equipment, and essential personnel were clear themes that emerged from the data. Notably, the most discussed aspect of PHC satisfaction was not satisfaction of the medical treatment but behavior of the staff. Many participants reported feeling greater levels of trust in and satisfaction with PHC services when they believed their caretakers had good bedside manner. As a corollary, participants reported stronger feelings of distrust and lower perceived treatment quality when they perceived their caretaker's attitudes as poor. Therefore, in addition to increasing resources and investment in PHC, we recommend more research into the influence of bedside manner and the patient-physician relationship in PHC prevention and management of CMDs in Nepal, and how this can be improved

All participants reported their PHC lacked needed resources to provide them with appropriate care. This drove interest in seeking care at distant tertiary facilities and from specialists in cardiovascular medicine. Based on existing evidence, we suggest one potential solution to the challenges identified in our study is electronic health (“e-health”) innovations such as mobile health and telemedicine interventions. E-health provides a platform to deliver some critical aspects of CMD care directly to people in their own hometowns, even in the most isolated and rural areas (42–44). Over 3/4 of Nepali households use a mobile phone (45) and previous studies have reported encouraging results with e-health interventions in the control of CMDs both in Nepal (46–48) and other countries (49, 50). While the potential of these interventions is clear, implementation is a challenge in resource-limited settings. A recent review of e-health interventions in Nepal found that many were not adequately integrated into the existing healthcare system nor scaled beyond a local level (51). Therefore, we suggest this may also be a worthwhile target for future research aimed at improving CMD control in Nepal.

## Conclusion

This study used mixed-methods to investigate use and perception PHC services for CMDs in two low-resource settings in Nepal. PHC use was high but overall satisfaction was low. Medication cost was the strongest point of dissatisfaction, with 100% of interview participants recommending increasing medicine availability and 85% suggesting to decrease medicine cost. Perceptions of provider competency were tied to the perceived quality of their bedside manner. Therefore, we suggest bedside manner and the patient-physician relationship are important and practical targets for future studies aimed at improving PHC-level CMD control in this setting. Finally, we identified several key barriers to PHC use, including: financial challenges, lack of equipment/essential medications, and geographic barriers. While we did not directly investigate e-health, based on existing evidence, we suggest e-health interventions may be a complementary, potentially effective strategy to mitigate the barriers to PHC services identified in our study. Therefore, we propose this is an additional area for further research.

As stated in 2019 by (21) “Primary care is more than a first point of care; it is the core process of a health system”. We strongly recommend further efforts aimed at enhancing the prevention and management of CMDs at the PHC level in Nepal, which will contribute to overall health system strengthening.

## Data Availability Statement

The raw data supporting the conclusions of this article will be made available by the authors, without undue reservation.

## Ethics Statement

The studies involving human participants were reviewed and approved by Duke University Institutional Review Board (Protocol Number: Pro00082962) Nepal Health Research Council (NHRC) (Reg. No. 158/2017). The patients/participants provided their written informed consent to participate in this study.

## Author Contributions

NP, EG, KG, SNK, BAK, SK, AA, and LY conceived the study design and contributed substantially to data acquisition, analysis, and interpretation, and production and approval of the final manuscript. SX, TØ, JM, and QL contributed substantively to data analysis and interpretation, critical revision, and final approval of the manuscript. All authors contributed to the article and approved the submitted version.

## Funding

This study was funded by the World Health Organization Asia Pacific Observatory on Health System and Policies (supporting the overall activities of the four country FAITH study, of which this study is a part): WHO Registration: 2016/622444-0; grant number: 2014/77862. Duke University and Duke Kunshan University provided financial support to graduate students who conducted fieldwork in Nepal for data collection.

## Conflict of Interest

The authors declare that the research was conducted in the absence of any commercial or financial relationships that could be construed as a potential conflict of interest.

## Publisher's Note

All claims expressed in this article are solely those of the authors and do not necessarily represent those of their affiliated organizations, or those of the publisher, the editors and the reviewers. Any product that may be evaluated in this article, or claim that may be made by its manufacturer, is not guaranteed or endorsed by the publisher.

## Appendix

**Table A1:** Sociodemographic Profile of In-Depth Interview Participants.

**Participant ID**	**Location**	**Gender**	**Age**	**Occupation**	**CVD Condition**
**MS**	Urban	Male	61	Yoga Trainer	Heart Disease
KC	Urban	Male	67	Agriculture	Heart Disease, HTN
MJ	Urban	Male	49	Unemployed	Stroke, HTN
BD	Urban	Female	52	Agriculture	Stroke, HTN, Heart Disease
DV	Urban	Male	39	Agriculture	Stroke, HTN
JF	Urban	Female	54	Retired	HTN
AL	Urban	Female	58	Housewife	Heart Disease
**HS**	Urban	Male	52	NGO	HTN, Diabetes
TR	Urban	Male	43	Business	Stroke
**DN**	Urban	Female	41	Government	Heart Disease, Diabetes
BK	Rural	Male	75	Unemployed	HTN, Diabetes
MD	Rural	Female	64	Agriculture	HTN
SK	Rural	Female	26	Agriculture	Stroke, Heart Disease, HTN
KG	Rural	Male	37	Business	HTN, Diabetes
**JR**	Rural	Male	53	Government	HTN
ST	Rural	Female	35	Housewife	HTN
NP	Rural	Male	63	Unemployed	Heart Disease, HTN, Diabetes
LJ	Rural	Male	65	Retired	Stroke, HTN, Diabetes
**SS**	Rural	Female	71	NGO	Heart Disease
AT	Rural	Male	40	Tailor	HTN, Diabetes
